# Targeting Mitochondria in Glioma: New Hopes for a Cure

**DOI:** 10.3390/biomedicines12122730

**Published:** 2024-11-28

**Authors:** Lidia Gatto, Vincenzo Di Nunno, Anna Ghelardini, Alicia Tosoni, Stefania Bartolini, Sofia Asioli, Stefano Ratti, Anna Luisa Di Stefano, Enrico Franceschi

**Affiliations:** 1 Nervous System Medical Oncology Department, IRCCS Istituto delle Scienze Neurologiche di Bologna, 40139 Bologna, Italy; dinunnovincenzo88@gmail.com (V.D.N.); a.tosoni@isnb.it (A.T.); stefania.bartolini@ausl.bologna.it (S.B.); e.franceschi@isnb.it (E.F.); 2 Department of Medical and Surgical Sciences, University of Bologna, 40126 Bologna, Italy; anna.ghelardini@icloud.com; 3 Department of Biomedical and Neuromotor Sciences (DIBINEM), University of Bologna, 40126 Bologna, Italy; sofia.asioli3@unibo.it; 4 IRCCS Istituto delle Scienze Neurologiche di Bologna, 40139 Bologna, Italy; 5 Cellular Signalling Laboratory, Anatomy Center, Department of Biomedical Sciences (DIBINEM), University of Bologna, 40126 Bologna, Italy; stefano.ratti@unibo.it; 6 Division of Neurosurgery, Azienda USL Toscana Nord Ovest, Spedali Riuniti di Livorno, 56121 Livorno, Italy; annaluisadistefano@gmail.com; 7 Department of Neurology, Foch Hospital, 92150 Suresnes, France

**Keywords:** mitochondria, ONC201, OXPHOS, Warburg, metformin, IACS-010759

## Abstract

Drugs targeting mitochondrial energy metabolism are emerging as promising antitumor therapeutics. Glioma treatment is extremely challenging due to the high complexity of the tumor and the high cellular heterogeneity. From a metabolic perspective, glioma cancer cells can be classified into the oxidative metabolic phenotype (mainly depending on mitochondrial respiration for energy production) and glycolytic phenotype or “Warburg effect” (mainly depending on glycolysis). Herein, we reviewed the function of novel bio-active molecules targeting oxidative phosphorylation (OXPHOS), mitochondrial membrane potential and mitochondrial dynamics. These molecules exhibit intriguing preclinical and clinical results and have been proven to be promising candidates to be further developed for glioma therapy. However, despite these initial encouraging results, it is imperative to rigorously assess the side effects of these metabolic drugs, which have a non-negligible toxicity profile.

## 1. Introduction

Gliomas are infiltrative tumors of the central nervous system (CNS) that originate from glial cells and account for about 50–60% of intracranial tumors. Glioblastoma (GB) is the most common and aggressive primary intra-axial tumor in adults with an incidence of disease that progressively increases over the years [1,2]. Although it is a rare neoplasm, its incidence is approximately 3 people per 100,000 [3] and its prognosis is poor, with average patient survival of only 12–24 months from diagnosis [2]. GB still represents one of the most complex challenges in neuro-oncology today. Although progress in understanding the molecular biology of this tumor has been exciting, the therapeutic and prognostic implications remain disappointing.

The present standard of care includes maximal surgical removal of the tumor followed by treatment with temozolomide and radiotherapy [4]. Nevertheless, despite intense efforts, outcomes have improved only modestly: treatments do not effectively halt tumor progression and the outlook from diagnosis remains extremely poor. Therefore, the identification of novel therapeutic targets is urgently required and various studies have been undertaken in order to find novel agents for effective GB treatment, as well as modern anticancer drug delivery strategies [5,6]. In recent years, studies have suggested that targeting mitochondria could be a new strategy for intervention and therapy. Mitochondria are essential cellular organelles provided by their own genome (mitochondrial DNA) and delimited by a double membrane system, external and internal (mitochondrial cristae) delimiting and intermembrane space and internal matrix. They are involved in numerous biochemical pathways, including cellular metabolism regulation, redox signaling, energy generation, cell proliferation and apoptosis [7,8,9]. The leading pathway of mitochondrial metabolism is oxidative phosphorylation (OXPHOS), by which cells use enzymes to oxidize nutrients, ultimately converting the chemical energy to adenosine triphosphate (ATP), the source of energy for use and storage at the cellular level [10].

Mitochondria in cells may organize into interconnected cytoplasmic networks subject to structural alterations in a continuous process termed mitochondrial dynamics [11].

**Figure 1:**
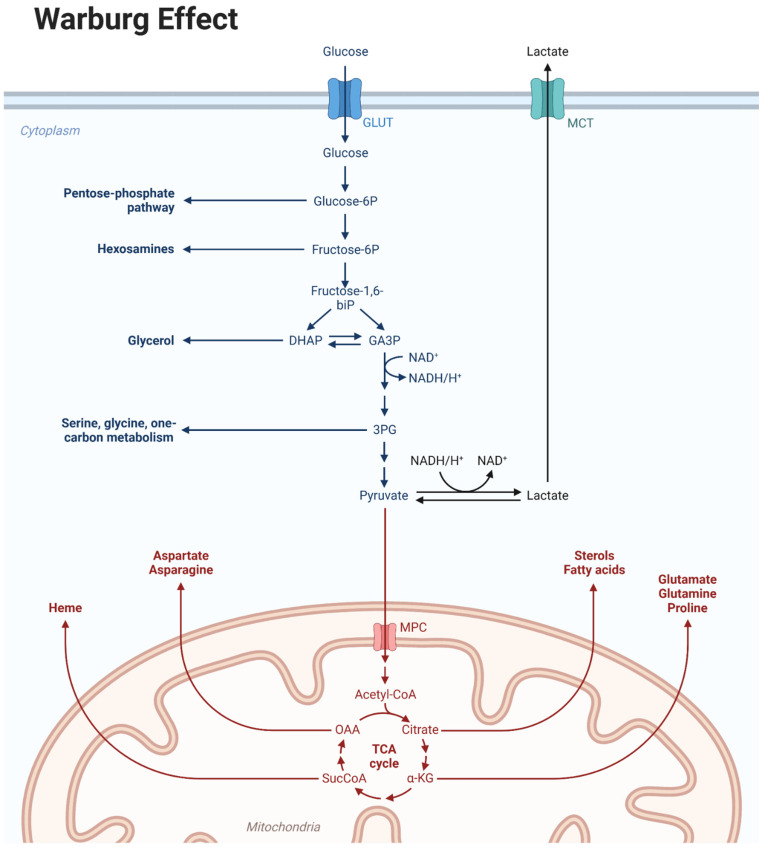
Warburg effect.

Metabolic reprogramming is a hallmark of GB and contributes to drug resistance [12,13]: GB continually readjusts its cellular metabolism to promote cellular plasticity, adapting to the availability of nutrients and acquiring increasingly aggressive characteristics. One of the distinctive characteristics of GB is a metabolic shift that provides survival advantage to tumor cells through their adaptation to aerobic glycolysis, with high glucose uptake, low oxygen consumption and high lactate production [12,14]. This phenomenon is characterized by increased tumor cell glycolysis and decreased mitochondrial energy metabolism, even in the presence of oxygen, with increased lactate production [15,16,17]. Aerobic glycolysis was observed for the first time in 1924 by Warburg [18,19,20,21], who suggested that this phenomenon, the “Warburg effect”, was the result of a mitochondrial dysfunction that prevents the complete oxidation of pyruvate in the mitochondria (Figure 1). Non-cancerous cells metabolize the final product of the glycolytic pathway, pyruvate, in the mitochondria, through the Krebs cycle and oxidative phosphorylation, a metabolic pathway that is particularly advantageous from an energetic point of view since it leads to the production of 36 molecules of ATP per molecule of metabolized glucose. Conversely, the typical glycolytic profile of cancer cells leads to a low production of ATP for each glucose molecule metabolized. Therefore, cancer cells tend to upregulate glucose transporters belonging to the GLUT family to significantly increase glucose absorption in an attempt to achieve an adequate energy yield [17] (Figure 1).

The Warburg effect describes a phenomenon in which cancer cells display increased glucose uptake and preferentially utilize glycolysis, even in the presence of oxygen. This metabolic shift towards glycolysis, rather than oxidative phosphorylation, provides cancer cells with the necessary energy and metabolic intermediates for rapid proliferation.

At the beginning of gliomagenesis, the transformed cells rely on anaerobic glycolysis alone for the production of ATP, as they are in a hypoxic microenvironment. Under these conditions, the Hypoxia-Inducible Factor-1 (HIF-1) progressively promotes the development of intra- and peritumoral vascularization [22]. Although tumors become vascularized over time thanks to this process of neo-angiogenesis, the glycolytic profile persists [23]. Recent evidence suggests that the glycolytic phenotype offers resistance to the process of apoptosis: many of the enzymes involved in glycolysis are in fact also important regulators of apoptosis, including hexokinase (HK). Although most glioma cells primarily rely on glycolysis for energy production, lactic acidosis, afterwards, progressively reduces glycolysis and significantly increases (approximately 2–4 times) OXPHOS processes to generate ATP. This is the reason why OXPHOS inhibitors can exhibit antitumor activity even in glycolytic glioma cells, so that the anticancer spectrum of OXPHOS inhibitors can be greatly expanded [24].

From a metabolic point of view, GB can be classified mainly into three groups: OXPHOS GB, which mainly uses oxidative phosphorylation as an energy source, “glycolytic” GB, which exploits the fermentative processes, and a third group without a specific metabolic predominance [25]. The first report on OXPHOS GB was from Frattini et al., who specifically studied the energetic metabolism of GB with Fibroblast Growth Factor Receptor 3 (FGFR3)-Transforming Acidic Coiled–Coil-Containing Protein 3 (TACC3) fusion, also known as F3T3 fusion [26]. The authors showed that F3T3 fusion GB exhibited an energetic metabolism mainly based on cellular respiration and OXPHOS pathways and had a specific vulnerability to antimitochondrial therapy [26,27].

The identification of metabolic vulnerabilities and their targeting in GB provides a promising approach to overcome disease progression and resistance to standard therapy. Nevertheless, it is important to underline that GB has a high intratumoral heterogeneity; thus, oxidative and glycolytic phenotypes coexist within the same tumor mass [28]. For example, recent studies have shown that the core of the tumor is glycolytic, whereas the periphery is more oxidative [29,30]. On the other hand, an aspect that should not be underestimated is the possibility that the standard therapies for GB, radiotherapy and chemotherapy, can alter the metabolic pathways, contributing to a “metabolic switch” of the tumor: this implies that untreated GB at onset may have a different metabolic signature from relapsed GB, already treated. These findings have important implications for the combination of treatments with different metabolic actions, for example, anti-OXPHOS + anti-glycolysis agents, in order to overcome intratumoral heterogeneity.

## 2. Identification of Mitochondrial GB and Therapeutic Implications

About 3% of GBs harbor FGFR-TACC fusions that can be identified by RT-PCR-sequencing and are mutually exclusive with Isocitrate dehydrogenase 1 and 2 (IDH1/2) mutations and epidermal growth factor receptor (EGFR) amplification whereas co-occur with Cyclin-Dependent Kinase 4 (CDK4) amplification. The FGFR-TACC fusion protein displays oncogenic activity, capable of increasing the number and activity of mitochondria [26,31]. Thanks to this mechanism, GB cells have a greater amount of energy available, which is essential for the uncontrolled multiplication and spread of tumor cells. The FGFR-TACC oncogene activates a protein called Peptidylprolyl Cis/Trans Isomerase, NIMA-interacting 4 (PIN4), which acts on peroxisomes, small cellular organelles, which normally metabolize fats and produce fuel for mitochondrial activity. The number of peroxisomes increases 4–5 times after the activation of PIN4 by FGFR-TACC fusion; in addition, their metabolic activity increases, causing the accumulation of oxidizing substances in the cell [26,31]. These substances stimulate the expression of PGC1-alpha, the master regulator for mitochondrial metabolism, which therefore becomes free to uncontrollably stimulate the activity of the mitochondria and energy production essential to GB. Via the FGFR-PIN4 pathway, FGFR-TACC-fused GB acquires a prevalent OXPHOS metabolism sustained by oxidative phosphorylation and mitochondrial respiration.

The FGFR/TACC fusion is also present, with percentages similar to that of GB, in other human tumors such as lung, esophageal, bladder, breast, cervical and head and neck cancer and it is probably the most frequent gene fusion described so far in cancer [26,31].

Iavarone and colleagues [27,32] clustered GB subtypes on the basis of neurodevelopmental and metabolic axes, providing a new GBM classification that identifies four functional groups that embody metabolic (mitochondrial and glycolytic/plurimetabolic) and developmental (neuronal and proliferative/progenitor) attributes and is capable of predicting prognosis and therapeutic vulnerabilities.

Mitochondrial GB [32], which is selectively dependent on OXPHOS for energy production and survival, exhibits a marked sensitivity to drugs inhibiting OXPHOS and mitochondrial functions in vitro and in vivo. In contrast, glycolytic/plurimetabolic GB, which is sustained by the activation of multiple interconnected metabolic pathways (aerobic glycolysis plus anabolism of lipids and amino acids), escapes mono-metabolic targeting [27,32].

Single-cell RNA-sequencing analysis of 232 GB patients confirmed this classification, clustering patients into molecular subgroups as follows: n = 60 OXPHOS (26%), n = 48 neuronal (20%), n = 55 proliferative (24%), n = 58 glycolytic (25%) and n = 11 unclassifiable (5%) [25]. F3T3 gene-fused samples showed that the IDH WT molecular profile has a clinically significant better survival and OXPHOS metabolism; thus, this rare subgroup can most likely benefit from mitochondrial inhibitors [25]. The reasons behind the better clinical outcome of mitochondrial GB are still not fully explained, but this subtype produces higher levels of reactive oxygen species and seems to be more sensitive to irradiation.

Patients with mitochondrial GB could benefit from a new therapeutic approach: several studies have shown that drugs inhibiting mitochondria have a powerful antitumor effect against GB cells with hyperactive mitochondria.

Iavarone et al. tested the sensitivity of 13 mitochondrial and 10 glycolytic/plurimetabolic GBs to compounds that interfere with OXPHOS and mitochondrial metabolism. They used four agents: two inhibitors of mitochondrial complex I (metformin [33] and IACS-010759), tigecycline, an inhibitor of mitochondrial protein translation, and menadione [34], an inducer of mitochondrial ROS and apoptosis [27]. These agents showed antitumor activity against mitochondrial tumor cells; conversely, the glycolytic/plurimetabolic GB cells were resistant to all four compounds [27].

Recently, methylation profiling emerged as a powerful tool for the identification of novel tumor entities in neuro-oncology. Tumor methylation profiles are presumed to reflect both the cell of origin and the modifications induced by the oncological transformation. A methylation-based classifier of central nervous system tumors (the DKFZ classifier) has been developed [35] and recognized by the current WHO classification of brain tumors. Wu et al. [36] identified a small subset of F3T3-positive high-grade gliomas with a histological low-grade look but molecular features of IDH-wildtype GB (chromosome 7 gain, chromosome 10 loss, TERT mutation). This outlier subgroup, renamed by the authors GB-F3T3-O, included supratentorial tumors with variable morphology, including some cases with typical GB histomorphology, and the remainder showed oligodendroglioma-like morphology with calcifications and an absence of necrosis. GB-F3T3-O exhibited a distinct methylation profile characterized by a slightly increased global DNA methylation compared to other GBs, a predominant OXPHOS metabolism and better patient outcome compared to conventional GBs (median overall survival around 40 months).

## 3. Targeting Mitochondria in Glioma: Who to Recommend This Strategy to?

**Figure 2:**
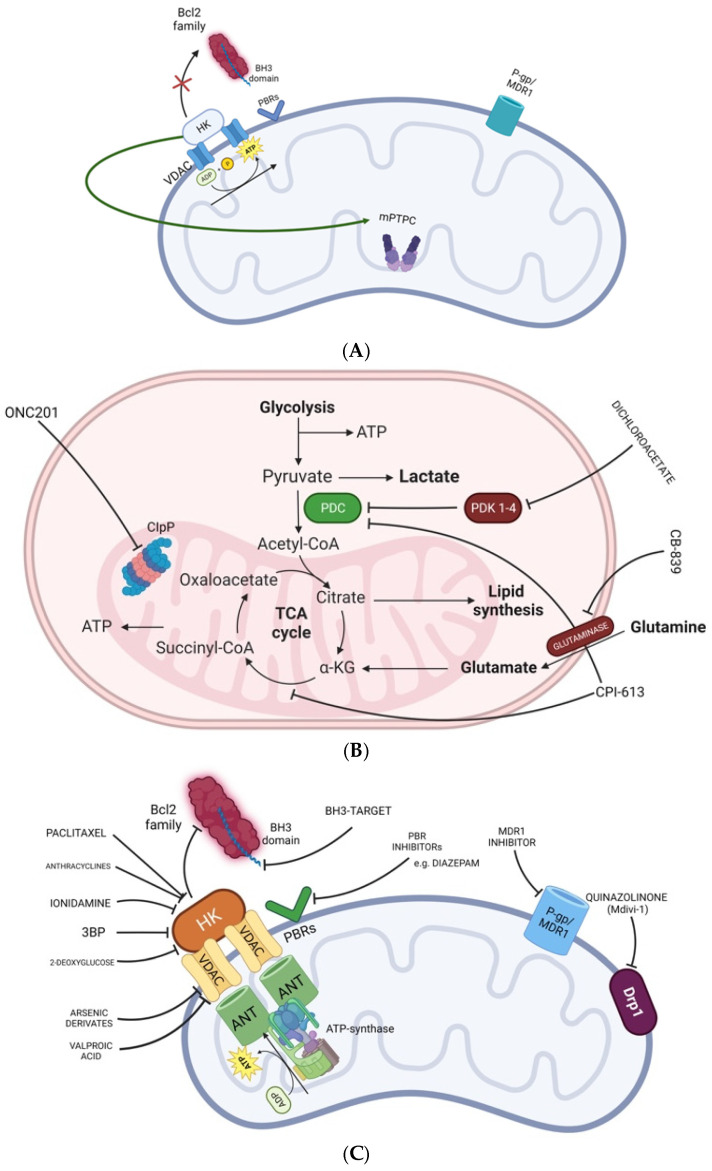
Small molecule mitochondrial metabolism inhibitors in pre-clinical or clinical development for the treatment of cancer, targeting (**A**) internal membrane, (**B**) matrix and (**C**) external membrane.

“Mitochondrial inhibitor” is a term grouping together a very large class of compounds acting at different sites and interfering with different pathways occurring in mitochondria (Table 1, Figure 2).

A first classification, for example, can be made considering the site at which inhibitors act: the outer mitochondrial membrane, the inner mitochondrial membrane, the mitochondrial matrix, the mitochondrial permeability transition pore complex (mPTPC) (Figure 2A–C), or the voltage-dependent anion channel. Mitochondria are not static organelles [37] characterized by dynamic transformations of the mitochondrial architecture and localization inside the tumor cell. Precisely, fusion (two or more mitochondria fuse in one tubular structure) and fission (mitochondria break down to single organelles with spatial distribution within the cytosol) are the essential forces of mitochondria dynamics [37,38]. Mitochondrial fusion optimizes the energetic efficiency of the cell while fusion inhibition promoting fission leads to a fragmented mitochondrial network, increased reactive oxygen species and unbalanced redox homeostasis, representing an overall stress response.

**Table 1:** Mitochondrial inhibitors, mechanisms of action, study results and adverse events.

Agent	Mechanism of Action	Level of Evidence	Study Results	Adverse Events
Metformin	Complex I inhibitor	phase I lead-in to a phase II factorial study [39]	the median OS was 21 months (95% CI, 16.2–29.7 months) and the 2-year survival rate was 43% the median OS was 21 months (95% CI, 16.2–29.7 months) and the 2-year survival rate was 43% the median OS was 21 months (95% CI, 16.2–29.7 months) and the 2-year survival rate was 43% (95% CI, 34–56%). Metformin plus Mefloquine, Memantine and Temozolomide demonstrated a median survival of 21 months with a 2-year OS of 43%	Lymphopenia was the most common adverse event and the most common of all grade 3 and 4 adverse events lymphopenia was the most common adverse event and the most common of all grade 3–4 events
IACS-010759	Complex I inhibitor	phase I clinical trial [40]	IACS-010759 did not meet the primary objective of the study, ORR	optic neuropathy; severe visual impairment; hand/feet and/or leg/hip peripheral neuropathy; myalgia; weakness
Gboxin	Complex V inhibitor	in vitro studies [41]	Gboxin inhibits the growth of primary mouse and human GB cells, compromising oxygen consumption	/
Gamitrinib	Mitochondrial matrix inhibitor	in vitro and in vivo evidence [42]	Gamitrinib plus BH3 mimetics prolong survival in an orthotopic glioma patient-derived xenograft model	no detectable noxious effects on solid organs
ONC201	Allosteric agonist of CLPP	phase II study [43]	Median PFS of 14 weeks and median OS of 17 weeks in H3 K27M-mutant diffuse midline glioma	no dose-limiting toxicities, dose modifications or treatment discontinuation due to drug-related toxicity occurred in any patient
BH3-mimetics	External mitochonrial membrane inhibitor	in vitro and in vivo evidence [44]	ABT-737 exhibits single-agent-mechanism-based killing of cells from solid tumors and in animal models improves survival	no detectable noxious effects on solid organs
CPI-613 (devimistat)	TCA cycle inhibitor targeting the pyruvate dehydrogenase and the α-ketoglutarate dehydrogenase	(a) phase III study AVENGER 500 [45] (b) phase II study [46]	CPI-613 in combination with chemotherapy did not improve survival in pancreatic cancer compared with chemotherapy alone CPI-613 in combination with high-dose cytarabine and mitoxantrone did not meet the primary objective of the study, i.e., to determine if the maintenance schedule of CPI-613 was feasible	no new toxicity signals with the addition of CPI-613 no unexpected toxicities observed

Divergent data have been reported on features of OXPHOS tumors in oncology so far [47,48,49,50]; however, the common denominator is represented by sensitivity to antimitochondrial drugs [51] such as biguanides and newer more potent inhibitors.

The first obstacle to consider for targeting mitochondria in GB is the remarkable heterogeneity of glioma tumor cells, which express a different grade of dependence from mitochondrial activity [27,32,52]. The mitochondrial GB subgroup exhibits the almost exclusive mutation of the NRAS gene and the deletion of the Solute Carrier Family 45 Member 1 (SLC45A1) gene [53]. This gene encodes for a glucose–proton symporter whose activity leads to a further reduction in intracellular pH. The NRAS mutations as well as SLC45A1 deletions could be two elements essential to recognizing mitochondrial GB as more sensitive to mitochondrial inhibitors to pursue a precision medicine approach [53].

In the past, the lack of distinction between GB with different metabolic phenotypes could have impacted negatively on the results of clinical trials testing mitochondrial inhibitors. For example, in 2003, the combination of lonidamine and diazepam (acting on two distinct mitochondrial mechanisms) was assessed in a phase II study for recurrent GB that failed to show convincing clinical benefit except for disease stabilization [54].

Clinical trials assessing mitochondrial inhibitors should now take into consideration the identification of GB subgroups more likely to respond [55,56].

Within GB, a rare subgroup of gliomas seems to show a high sensitivity to the inhibition of a mitochondrial proteinase. This subgroup of tumors is named diffuse midline glioma (DMG) and is characterized by the absence of IDH mutations (IDH wild-type tumors) and by the presence of histone 3 (H3) alterations [57]. A novel class of drugs also targeting the mitochondrial Caseinolytic Protease P (CLPP) seems likely to be capable of changing the clinical course of the disease [57]. However, the exact metabolic mechanism behind this apparent sensitivity is still largely obscure.

## 4. Compounds Targeting the External Mitochondrial Membrane

Hexokinase (HK) catalyzes the first step of glycolysis. The HK isoform 2 is linked to the external mitochondrial membrane, which can capture adenosine triphosphate (ATP) from the voltage-dependent anion channel (VDAC) and can also inhibit the pro-apoptotic BcL-2 family proteins [58]. Finally, HK can activate the mitochondrial permeability transition pore (MPTP) complex. HK inhibitors include lonidamine as well as 3 bromopyruvate (3BP) and 2-deoxyglucose. None of them showed clinical efficacy in gliomas [59].

The Bcl-2 family proteins provide pro- and anti-apoptotic stimuli. The BH3 domain of these proteins is essential to drive Bcl-2 toward a pro-apoptotic activation. Thus, agents targeting BH3 and promoting the apoptosis process are of particular interest [60] (Table 1, Figure 2C). However, none of these drugs is currently under investigation in glioma patients. Peripheral benzodiazepine receptors (PBRs) are mitochondrial membrane proteins largely expressed by astrocytes and microglia (especially in steroid-producing cells) (Figure 2C). These receptors play important biological roles acting on apoptosis control, stress response, microglia activation, steroid synthesis and the regulation of mitochondrial membrane potential [61,62]. The development of inhibitors of benzodiazepine receptors (Figure 2C) appears of particular interest for glioma treatment but also for neurological disorders. Diazepam is an inhibitor of PBR and has been tested with lonidamine in recurrent GB failing to show convincing clinical outcomes [54].

Finally, mitochondrial P-gp (MDR1) is a channel protein able to drive drug efflux outside the cell and thus mitochondria. P-gp (MDR1) inhibitors could make tumoral cells sensitive to chemotherapy, including temozolomide [63] (Figure 2C).

## 5. Compounds Targeting Complexes I–V

**Figure 3:**
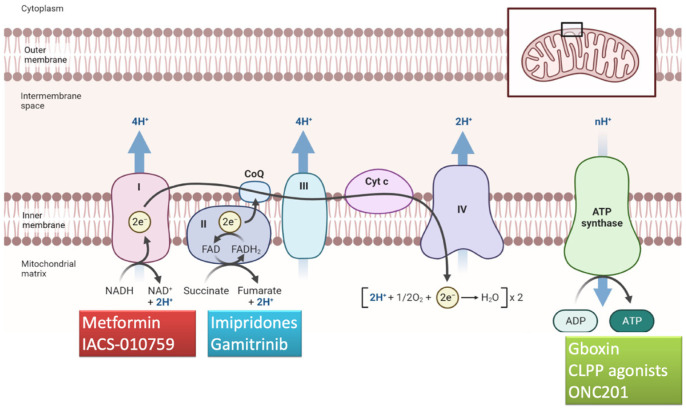
The electron transport chain is a crucial process in cellular respiration that occurs within the mitochondria. It involves a series of protein complexes (I–V), where high-energy electrons (2e^−^) are shuttled along the complexes, generating an electrochemical gradient that releases energy and produces ATP. Several inhibitors are highlighted that target complex I (metformin and IACS-010759), predominantly complex II (gamitrinib) and several complexes (Gboxin, imipridones, CLPP activators).

The electron transport chain is composed of the complexes I-IV and the ATP synthase (complex V) (Figure 3). These enzymes provide the central function of mitochondria, essential to provide the energy to the cell as ATP (Figure 2A,B and Figure 3). Electron transport through the respiratory chain is also associated with the production of reactive oxygen species (ROS), a physiological product of mitochondrial respiration [64].

Drugs stimulating or dysregulating the electron transport chain may mediate an over-production of ROS, unbalancing the redox homeostasis and activating apoptosis. Each respiratory complex has one or more inhibitors [65,66,67,68,69].

Complex I (NADH-ubiquinone oxidoreductase) inhibitors exhibit significant antitumor activity in glioma and include molecules with different biochemical structures but with similar activities, e.g., rotenoids, vanilloids, metformin and a novel compound called IACS-010759, which appeared to suppress the levels of the amino acid aspartate, pivotal for pyrimidine synthesis [10,70,71]. Metformin, a biguanide used as anti-diabetic drug, is an inhibitor of complex I that acts an OXPHOS inhibitor (Figure 3). Through the disruption of the mitochondrial-dependent ATP process, the downregulation of PI3K/mTOR pathway and the simultaneous activation of AMP-activated protein kinase (AMPK) phosphorylation, it is possible to increase cellular stress and to arrest the cell cycle, leading to the GB cells’ death [72,73,74]. Metformin has been investigated with mefloquine, memantine and temozolomide in GB patients after radiation treatment [39] in a phase II study that suggests effectiveness (median survival of 21 months with a 2-year OS of 43%) and a safety profile of the combined treatments.

OPTIMUM (NCT04945148) is an open label, non-randomized multicenter phase II trial investigating metformin in association with the standard first-line treatment with radiotherapy and temozolomide to treat patients affected by IDH wild-type GBs specifically dependent on the oxidative phosphorylation, detected by molecular analysis including RNA assay. The investigators expect to screen 640 patients and to include 64 patients over a period of 24 months with 24 months of follow-up.

IACS-010759 is a novel high-affinity OXPHOS inhibitor targeting mitochondrial complex I and induces apoptosis in models of brain cancer and acute myeloid leukemia (AML) [75].

Yap et al. [40] published the results of a phase I trial evaluating IACS-010759 in individuals with acute myeloid leukemia and solid tumors. Unfortunately, promising preclinical findings did not translate into the clinic because of intolerable neurotoxicity that precluded adequate dosing. In multiple preclinical models [76] of glycolysis-deficient tumors, IACS-010759 extended mouse survival [77,78], and was shown to be well tolerated, with no observed neurotoxicity. Based on these preclinical findings, two first-in-human phase I clinical trials [40] were started. The first trial evaluated patients with relapsed/refractory acute myeloid leukemia (AML), a malignancy strongly dependent on OXPHOS [40]. The second trial (NCT03291938), instead, enrolled patients with glycolysis-deficient advanced solid tumors. Elevated blood lactate associated with persistent and dose-dependent peripheral and optic neuropathy emerged as prominent side effects in both trials, with peripheral neuropathy characterized by hand/foot and/or leg/hip numbness, myalgia and weakness observed in both the solid tumor and AML patients. In addition, one patient developed severe visual impairment, as a consequence of optic nerve toxicity [40]. Consequently, IACS-010759 plasma exposure was challenging to maintain due to the drug interruptions necessary to mitigate the adverse events and only limited antitumor activity was observed at tolerated doses. In fact, only 1 patient (out of 40) had an objective response. These “off-target” effects might be explained by the 1H-1,2,4-triazole contained in IACS-010759, which is a mitochondrial pantoxic motif [79]. Interestingly, the coadministration of an HDAC6 inhibitor demonstrated preventive action against IACS-010759-induced peripheral neuropathy in mouse models [40,78].

IM156, a novel biguanide, mitochondrial complex I inhibitor, was tested in a phase I study in 22 patients with refractory or advanced solid tumors [80]. No dose-limiting toxicities were observed and the most frequent treatment-related adverse effect was nausea. Stable disease was observed in about 30% of patients [80]. These results pave the way for further clinical development of IM 156.

Complex II (succinate dehydrogenase) inhibitors (*α*-tocopheryl succinate, gracillin and atpenins) induce mitochondrial ROS production and sensitize cells to apoptosis [10,81].

The complex III inhibitors Licochalcone A and antimycin A have been shown to increase apoptosis and reduce the viability of glioma stem cells [10,82]. Verteporfin, used as a photosensitizer for photodynamic therapy to eliminate the abnormal blood vessels in the eye associated with conditions such as the wet form of macular degeneration, has been characterized as an OXPHOS inhibitor in glioma stem cells [10,83]. Atovaquone, a drug used to prevent and treat pneumonia caused by the protozoan Pneumocystis carinii, is a competitive inhibitor of Complex III and is reported to be effective against cancer stem cells [84]. Arsenic trioxide, a Complex IV inhibitor, showed promising anticancer effects in glioma by apoptosis and autophagy of tumor stem cells, through the production of intracellular reactive oxygen species (ROS) [85].

Complex V synthesizes ATP from ADP in the mitochondrial matrix using the energy provided by the proton electrochemical gradient and plays an important role in GB metabolism [10] and survival [86]. Multiple Complex V inhibitors have shown promising anticancer potentials in vitro and in vivo [10]. Bedaquiline, an antibiotic member of the diarylquinoline class targeting the adenosine triphosphate (ATP) synthase enzyme of the tuberculosis mycobacteria, specifically targets Complex V, leading to apoptosis of stem-like cancer cells. Similarly to Bedaquiline, other molecules, like Gboxin [41], resveratrol, quercetin, tenoxin and lecucinostatin, also have the ability to inhibit ATP synthase [87], but the current knowledge of their activity against GB cells is still incomplete and controversial.

Gboxin is a well-known complex V inhibitor (Table 1, Figure 3) that specifically suppresses GBM cell proliferation [41], whose effectiveness is seriously limited by an extremely short half-life, poor blood circulation due to the blood–brain barrier and by non-specific GBM tissue/cell uptake, leading to insufficient Gboxin accumulation at GBM sites. Careful selection of mitochondrial inhibitor ligands and targeted delivery are essential to ensure greater selectivity of these therapeutic agents and to limit toxicity to healthy tissues. Zou et al. presented cancer cell–mitochondria hybrid membrane camouflaged nanomedicines with biomimetic nanoparticles for the targeted delivery of Gboxin [6]. This novel compound showed controlled Gboxin release under conditions that mimic the high ROS tumor environment, with enhanced mitochondria targeting and antitumor efficacy [6].

## 6. Compounds Targeting the Mitochondrial Matrix

The TCA cycle is an essential biochemical pathway occurring in the mitochondrial matrix. The inhibition of the pyruvate dehydrogenase kinase is a key aspect able to improve OXPHOS metabolism with a decreased glycolytic metabolism [69]. Devimistat, also known as CPI-613, specifically targets the TCA cycle, simultaneously inhibiting two TCA cycle enzymes, the pyruvate dehydrogenase and the α-ketoglutarate dehydrogenase, dramatically compromising mitochondrial metabolic flows and triggering multiple, redundant pathways in tumor cells [72,88]. CPI-613 showed impressive phase I results [30] (Table 1, Figure 2B); however, two phase III trials failed to demonstrate enhanced efficacy in pancreatic cancer and acute myeloid leukemia [78].

Gamitrinib, also known as geldanamycin, is a mitochondrial matrix inhibitor [70,89] that has shown efficacy in GB model systems, especially in association with BH3-mimetic [42,70]. It is currently undergoing assessment in a phase I clinical trial involving patients with advanced malignancies, including GB (Table 1) [72].

The mitochondrial permeability transition pore complex (mPTPC) is a channel that mediates the transport of ATP and ADP and is thought to initiate apoptosis (Figure 2A). Agents such as paclitaxel and anthracyclines have an apoptotic effect by mediating the opening of this channel (Figure 2C) [65].

The alterations of other metabolic pathways in the mitochondrial matrix, including the stimulation of estrogen receptors and fatty acid oxidation, are mainly assessed for drug development of metabolic and vascular/ischemic disease rather than for cancer treatments [90].

Thanks to the availability of a specific target, CLPP is gaining increasing interest [91]. This protein is essential to maintaining protein quality control in the mitochondria by degrading altered proteins [91]. The inhibition of this protease results in impaired oxidative phosphorylation, which mediates an anticancer effect. Some tumors including DMG may express a significant sensitivity to the inhibition of this protease [92,93,94]. It is still unclear if other gliomas could benefit from the inhibition of this complex.

## 7. Compounds Targeting the Mitochondrial Dynamics

Two other mitochondrial processes deserve interest in terms of their potential therapeutic implications: mitochondrial membrane potential and mitochondrial dynamics [10]. GB cells surprisingly exhibit hyperpolarized mitochondrial membrane potential, which allows the cancer cell to escape apoptosis and programmed cell death [41,95,96]. Inhibiting OXPHOS has the additional effect of depolarizing the mitochondrial membrane, which can secondarily promote cancer cell death. Mitochondrial dynamics is another emerging target for GB therapy; however, it needs to be further examined. The process of fusion and fission depends on different dynamin-related proteins which are mitofusin–1 (MFN1), mitofusin-2 (MFN2) and optic atrophy 1 (OPA1) for fusion and dynamin-1-like-protein (DPR1) and mitochondrial fission 1 protein (FIS1) for fission [97].

In solid tumors, including GB, mitochondria are often distributed in a fragmented pattern (unbalanced toward fission) due to a DRP1 upregulation [98]. Mitochondrial fission is associated with cell cycle progression, increased invasiveness and metastatic capacities.

Quinazolinone (Mdivi-1) is an inhibitor of DRP1 (Figure 2C) that showed promising results in preclinical data but no studies testing this agent are available [99]. Even if mitochondrial dynamics appears a very promising novel strategy for cancer treatment, currently no drugs directly targeting this pathway are available or under clinical trial assessment for glioma patients.

## 8. Menadione/Ascorbate Combination

New therapeutic strategies have been developed to modulate GB redox signaling to effectively suppress growth and prolong survival. The redox active combination of menadione and ascorbate has proved feasible and promising and deserves the attention of clinicians [100].

Menadione/ascorbate has been used in mouse GB and has demonstrated tumor growth suppression and survival advantage, as well as increased brain perfusion and decreased regulation of several oncoproteins and oncometabolites, which implies modulation of the immune response and reduced drug resistance [100].

## 9. ONC201 Targets Mitochondrial Metabolism in H3 K27-Mutant Midline Glial Tumors

ONC201 is an orally active small molecule belonging to the class of imipridones, with a unique mechanism of action: it selectively antagonizes the dopamine D2 receptor (DRD2), an important neurotransmitter, acts as an allosteric agonist of CLPP (Table 1, Figure 2B and Figure 3) and upregulates apoptotic factors such as the TNF-related apoptosis-inducing ligand (TRAIL). DRD2 controls growth factor signaling and promotes tumor growth and has emerged as a therapeutic target for gliomas and other tumors that overexpress this receptor [43,101]. Interestingly, DRD2 is overexpressed in GB and in midline brain tumors: a DRD2 blockade is sufficient to inactivate growth factor signaling and induce apoptosis; thus, these tumors have enhanced sensitivity to DRD2 antagonists [43]. Furthermore, ONC201 hyperactivates CLPP, leading to selective degradation of mitochondrial proteome components and subsequent activation of the integrated stress response and apoptosis [102,103].

ONC201 crosses the blood–brain barrier and has demonstrated anticancer activity and apoptosis-like effects in a range of difficult-to-treat cancer forms, including H3K27-mutant gliomas, by targeting dopamine receptors and mitochondrial metabolism [104].

The mechanisms by which ONC201 inhibits cell growth or induces cell death remain to be determined and no defined mechanism of action has been established for ONC201 [105,106,107]. The rapid clinical advance of ONC201 has preceded a detailed understanding of the molecular mechanism of drug action. The existing literature points to multiple pathways, e.g., tumor necrosis factor (TNF)-related apoptosis-inducing ligand (TRAIL) signaling, dopamine (DRD2) receptor antagonism and mitochondrial metabolism, as putative drug targets; nevertheless, mitochondrial metabolism seems to be the major target of imipridones [108]. Indeed, it induces changes in mitochondrial morphology, the inhibition of oxidative phosphorylation and increased lactic acid formation.

In November 2018, the FDA granted fast-track designation to Oncoceutics’ investigational cancer drug ONC201 for the treatment of adult recurrent H3 K27M-mutant high-grade glioma to facilitate the development of this agent from translational research into a clinical development program and to test its activity in clinical trials.

The Phase I study investigating ONC201 was conducted in 2017 and included 10 heavily pretreated patients with refractory solid tumors and an additional 18 patients with advanced disease; no glioma patients were included [109]. This study demonstrated that the compound was well tolerated and set the recommended dosage at 625 mg orally, once every 3 weeks.

The first phase II trial (NCT02525692) with GB came in the same year by Arrillaga-Romany et al. [110], who included 17 bevacizumab-naïve patients with progressive/recurrent GB and several poor prognosis features. Two patients remained on therapy for >12 months. Median OS was 41.6 weeks, OS at 6 months was 71% and OS at 9 months was 53%. No drug-related SAEs or treatment discontinuation due to toxicity occurred [110]. The kinetics of the response were unusual, exhibiting slow but sustained regressions of the lesions, similar to responses observed with immune checkpoint inhibitors, in line with the supposed immunomodulatory activity of ONC201 [110,111]. Although the study did not achieve the primary endpoint, PFS, the durable objective response observed in the patient harboring a H3.3 K27M mutation highlighted the relevance of this mutation to the activity of ONC201 and the need for further investigation. Following up on this durable response, an expanded access program entirely restricted to H3 K27M-mutant diffuse midline gliomas was initiated [43]. A total of 18 patients with H3 K27-mutant diffuse midline glioma were enrolled. Fourteen patients with recurrent disease and four pediatric patients following radiation, but prior to disease recurrence, participated in the study. Among the 14 patients with recurrent disease, median progression-free survival was 14 weeks and median overall survival was 17 weeks. Three adults among the 14 recurrent patients remained on treatment progression-free with a median follow-up of 49.6 (range 41–76.1) weeks. Among the four pediatric patients who initiated adjuvant ONC201 following radiation, two patients remained progression-free for at least 53 and 81 weeks. One adult with recurrent H3 K27M-mutant diffuse midline glioma exhibited a complete response: this 38-year-old patient was pretreated with radiotherapy, temozolomide and CCNU [43].

Hall et al. [112] reported clinical experience in a pediatric patient with H3 K27-mutant midline glioma enrolled in a compassionate use study. The 10-year-old girl presented with House–Brackmann grade IV facial palsy and unilateral hearing loss. The tumor volume sequentially decreased by 26%, 40% and 44% over 6 months, and remained stable at 18 months. Ipsilateral hearing normalized and the facial palsy improved to House–Brackmann grade I by 4 months [112].

Tanrikulu et al. [113] have published the results of a retrospective study with German-sourced ONC201 in 18 H3K27-altered pediatric pontine diffuse midline gliomas. With the limits due to the retrospective nature of this study, they reported considerably longer median OS in the ONC201 group (19.9 vs. 10.9 months).

The clinical outcomes and radiographic responses in these patients provide the preliminary and initial clinical proof-of-concept for targeting H3K27-mutant diffuse midline glioma with ONC201, regardless of age or location, providing rationale for robust clinical testing of the agent.

Recently, Arrillaga-Romany et al. [114] published the results of an integrated analysis that included patients from five clinical studies of ONC201 ([114], NCT03295396, NCT03416530, ONC016 single-patient compassionate use program [43]). Eligible patients were adult and pediatric patients affected by recurrent and/or progressive H3 K27M-mutant diffuse midline glioma with a Karnofsky score of ≥60, who had received previous radiation therapy with a washout of ≥90 days before the first ONC201 dose. The adults received ONC201 as oral capsules at a dosage of 625 mg/day. For the pediatric patients, the dose was calculated by body weight. ONC201 monotherapy was well tolerated and exhibited durable and clinically meaningful efficacy. In particular, the ORR was 30%, with a time to response of 8.3 months. In addition, a ≥50% corticosteroid dose reduction occurred in 47% of the evaluable patients. No grade 4 side effects, deaths or discontinuations occurred [114].

The double-blind, randomized, placebo-controlled ACTION trial (NCT05580562) is a phase III study in patients with newly diagnosed H3 K27M-mutant diffuse glioma to assess whether treatment with ONC201 following frontline radiotherapy can extend OS and PFS in this population. Eligible participants are adult and pediatric patients who have histologically diagnosed H3 K27M-mutant diffuse glioma and should have completed standard frontline radiotherapy. There are three arms: two experimental arms with different schedules of ONC201 and a third arm, the placebo arm. This study has a dual primary end point of OS and PFS. The study is actively recruiting and the estimated study completion date is August 2026.

## 10. OSMR Gene Knocking-Out

One of the reasons why GB is so difficult to attack lies in the particular type of stem cells involved. The OSMR gene supplies GB stem cells with energy and helps strengthen the resistance of cancer stem cells to radiotherapy by strengthening the mitochondrial function. Conversely, suppressing the OSMR gene increases response to radiotherapy and increases survival. Experiments on mice are promising [12]. In fact, it has been observed that the suppression of the OSMR gene, which controls energy production in GB stem cells, reduces the resistance of the tumor, making it more vulnerable to radiotherapy. Thus, targeting the OSMR gene has proven to be a successful strategy in experiments on mice. By knocking out the OSMR gene, significant improvements in the response to therapy were achieved, so much so that the survival of the animals increased significantly [12]. The next step will be to start a clinical trial to test whether the OSMR gene blackout strategy also works in humans.

## 11. Future Perspectives for Mitochondrial Inhibitors: The Road Ahead to Optimization

Metabolomics, a rapidly evolving field, has shed light on the intricate metabolic profile of GB, offering a promising avenue to the development of innovative potential therapeutic targets customized to the specific metabolic phenotype of this tumor [72]. Metabolic therapy should focus on both the glycolytic and oxidative subpopulations of GB [28]. To date, there are very few data about the role of mitochondrial inhibitors in gliomas. Understanding mitochondrial function in normal and tumor cells is crucial to address the risks and feasibility of mitochondrial inhibitors for anticancer therapy.

The toxic side effects reported in the phase I trial evaluating the complex I inhibitor IACS-010759 suggest that further research is necessary to elucidate the toxicities of these drugs on normal tissues, heavily dependent on the OXPHOS pathway. As complex I inhibitors continue to be evaluated as anticancer agents, there is an urgent need to critically and comprehensively assess their toxicity before advancing the clinical translation of these compounds [40,78].

Several ongoing trials with ONC201, a member of the imipridone class of anticancer small molecules targeting mitochondria through the activation of CLPP mitochondrial protease [115], are testing this agent in H3K27 patients, with encouraging results. Jackson et al. [116] have observed that H3K27-altered diffuse midline gliomas harboring PIK3CA-mutations have increased sensitivity to ONC201, while those harboring TP53-mutations are more resistant. Metabolic adaptation and reduced sensitivity to ONC201 are promoted by redox-activated PI3K/Akt signaling, which could be counteracted using the brain penetrant PI3K/Akt inhibitor paxalisib. Together, these discoveries, coupled with the powerful pharmacokinetic and pharmacodynamic properties of ONC201 and paxalisib, have provided the rationale for the ongoing phase II combination clinical trial NCT05009992. This phase II trial is aimed at determining if the combination of ONC201 with different drugs, i.e., panobinostat or paxalisib, is effective for treating patients with diffuse midline gliomas.

Furthermore, a novel synergistic combination therapy involves imipridones and HDAC inhibitors [70]. GB cells chronically exposed to HDAC blockers increase their oxygen consumption rate [70,117]. Because of the impact of HDAC inhibitors on metabolism, it has been hypothesized that imipridones, which suppress cellular respiration, might synergize with these compounds to significantly reverse the HDAC inhibitor-induced activation of cellular respiration, inducing GB cell death [70,118].

In light of the results of the studies conducted so far and considering the neurotoxicity profile of these compounds, the development of increasingly effective and better tolerated OXPHOS inhibitors is the goal to achieve to improve the treatment of GB. Nevertheless, given the intrinsic metabolic heterogeneity of GB, developing combined treatments that include both anti-OXPHOS and anti-glycolytic agents could be the way to best exploit the metabolic vulnerability of this tumor. Several anti-glycolytic agents, such as dimethylaminomicheliolide (DMAMCL) [119] and CPI-613 have demonstrated antitumor activity in glioma cells [72]; therefore, the study of cumulative toxicities of anti-OXPHOS and anti-glycolytic combos is, currently, a topic of great interest.

## References

[1] Kanderi T., Gupta V. (2003). Glioblastoma Multiforme. Statpearls.

[2] Louis D.N., Perry A., Wesseling P., Brat D.J., Cree I.A., Figarella-Branger D., Hawkins C., Ng H.K., Pfister S.M., Reifenberger G. (2021). The 2021 Who Classification of Tumors of the Central Nervous System: A summary. Neuro-Oncology.

[3] Ostrom Q.T., Cioffi G., Waite K., Kruchko C., Barnholtz-Sloan J.S. (2021). Cbtrus Statistical Report: Primary Brain and Other Central Nervous System Tumors Diagnosed in the United States in 2014–2018. Neuro-Oncology.

[4] Stupp R., Mason W.P., Van Den Bent M.J., Weller M., Fisher B., Taphoorn M.J., Belanger K., Brandes A.A., Marosi C., Bogdahn U. (2005). Radiotherapy Plus Concomitant and Adjuvant Temozolomide for Glioblastoma. N. Engl. J. Med..

[5] Rabha B., Bharadwaj K.K., Pati S., Choudhury B.K., Sarkar T., Kari Z.A., Edinur H.A., Baishya D., Atanase L.I. (2021). Development of Polymer-Based Nanoformulations for Glioblastoma Brain Cancer therapy and Diagnosis: An Update. Polymers.

[6] Zou Y., Sun Y., Wang Y., Zhang D., Yang H., Wang X., Zheng M., Shi B. (2023). Cancer cell-mitochondria hybrid membrane coated gboxin loaded nanomedicines for glioblastoma treatment. Nat. Commun..

[7] Nunnari J., Suomalainen A. (2012). Mitochondria: In Sickness and in Health. Cell.

[8] Gammage P.A., Frezza C. (2019). Mitochondrial Dna: The overlooked oncogenome?. BMC Biol..

[9] Guntuku L., Naidu V.G., Yerra V.G. (2016). Mitochondrial Dysfunction in Gliomas: Pharmacotherapeutic Potential of Natural Compounds. Curr. Neuropharmacol..

[10] Wu Z., Ho W.S., Lu R. (2022). Targeting Mitochondrial Oxidative Phosphorylation in Glioblastoma therapy. Neuromolecular Med..

[11] Tilokani L., Nagashima S., Paupe V., Prudent J. (2018). Mitochondrial Dynamics: Overview of molecular mechanisms. Essays Biochem..

[12] Sharanek A., Burban A., Laaper M., Heckel E., Joyal J.S., Soleimani V.D., Jahani-Asl A. (2020). Osmr controls glioma stem cell respiration and confers resistance of glioblastoma to ionizing radiation. Nat. Commun..

[13] Agnihotri S., Zadeh G. (2016). Metabolic reprogramming in glioblastoma: The influence of cancer metabolism on epigenetics and unanswered questions. Neuro-Oncology.

[14] Hsu P.P., Sabatini D.M. (2008). Cancer cell metabolism: Warburg and Beyond. Cell.

[15] Orzan F., Pagani F., Cominelli M., Triggiani L., Calza S., De Bacco F., Medicina D., Balzarini P., Panciani P.P., Liserre R. (2020). A simplified integrated molecular and immunohistochemistry-based algorithm allows high accuracy prediction of glioblastoma transcriptional subtypes. Lab. Investig..

[16] Leão Barros M.B., Pinheiro D.D.R., Borges B.D.N. (2021). Mitochondrial DNA Alterations in Glioblastoma (Gbm). Int. J. Mol. Sci..

[17] Ordys B.B., Launay S., Deighton R.F., Mcculloch J., Whittle I.R. (2010). The role of mitochondria in glioma pathophysiology. Mol. Neurobiol..

[18] Warburg O. (1956). On the Origin of Cancer Cells. Science.

[19] Bhattacharya B., Mohd Omar M.F., Soong R. (2016). The Warburg effect and drug resistance. Br. J. Pharmacol..

[20] Vaupel P., Multhoff G. (2021). Revisiting the Warburg effect: Historical dogma versus current understanding. J. Physiol..

[21] Vander Heiden M.G., Cantley L.C., Thompson C.B. (2009). Understanding the Warburg Effect: The metabolic requirements of cell proliferation. Science.

[22] Ramsay E.E., Hogg P.J., Dilda P.J. (2011). Mitochondrial metabolism inhibitors for cancer therapy. Pharm. Res..

[23] Gatenby R.A., Gillies R.J. (2004). Why do cancers have high aerobic glycolysis?. Nat. Rev. Cancer.

[24] Zeng S., Hu X. (2023). Lactic acidosis switches cancer cells from dependence on glycolysis to oxphos and renders them highly sensitive to oxphos inhibitors. Biochem. Biophys. Res. Commun..

[25] Di Stefano A., Picca A., Garofano L., Lerond J., Bielle F., Ducray F., Chinot O., Carpentier A., Younan N., Eoli M. (2023). Js04.7.A Analysis of RNA Classifies Newly Diagnosed Glioblastoma Patients and Identifies Patients Vulnerable To Targeted Metabolic therapies. Neuro-Oncology.

[26] Frattini V., Pagnotta S.M., Tala, Fan J.J., Russo M.V., Lee S.B., Garofano L., Zhang J., Shi P., Lewis G. (2018). A Metabolic Function of Fgfr3-Tacc3 Gene Fusions in Cancer. Nature.

[27] Garofano L., Migliozzi S., Oh Y.T., D’angelo F., Najac R.D., Ko A., Frangaj B., Caruso F.P., Yu K., Yuan J. (2021). Pathway-based classification of glioblastoma uncovers a mitochondrial subtype with therapeutic vulnerabilities. Nat. Cancer.

[28] Duraj T., García-Romero N., Carrión-Navarro J., Madurga R., Mendivil A.O., Prat-Acin R., Garcia-Cañamaque L., Ayuso-Sacido A. (2021). Beyond the Warburg Effect: Oxidative and Glycolytic Phenotypes Coexist within the Metabolic Heterogeneity of Glioblastoma. Cells.

[29] Darmanis S., Sloan S.A., Croote D., Mignardi M., Chernikova S., Samghababi P., Zhang Y., Neff N., Kowarsky M., Caneda C. (2017). Single-Cell RNA-Seq Analysis of infiltrating Neoplastic Cells at the Migrating Front of Human Glioblastoma. Cell Rep..

[30] Torrini C., Nguyen T.T.T., Shu C., Mela A., Humala N., Mahajan A., Seeley E.H., Zhang G., Westhoff M.A., Karpel-Massler G. (2022). Lactate Is an Epigenetic Metabolite That Drives Survival in Model Systems of Glioblastoma. Mol. Cell.

[31] Singh D., Chan J.M., Zoppoli P., Niola F., Sullivan R., Castano A., Liu E.M., Reichel J., Porrati P., Pellegatta S. (2012). Transforming fusions of FGFR and Tacc genes in human glioblastoma. Science.

[32] Lasorella A., Iavarone A. (2021). The making of the glioblastoma classification. Br. J. Cancer.

[33] Wheaton W.W., Weinberg S.E., Hamanaka R.B., Soberanes S., Sullivan L.B., Anso E., Glasauer A., Dufour E., Mutlu G.M., Budigner G.S. (2014). Metformin inhibits mitochondrial complex I of cancer cells to reduce tumorigenesis. eLife.

[34] Criddle D.N., Gillies S., Baumgartner-Wilson H.K., Jaffar M., Chinje E.C., Passmore S., Chvanov M., Barrow S., Gerasimenko O.V., Tepikin A.V. (2006). Menadione-induced reactive oxygen species generation via redox cycling promotes apoptosis of murine pancreatic acinar cells. J. Biol. Chem..

[35] Capper D., Jones D.T.W., Sill M., Hovestadt V., Schrimpf D., Sturm D., Koelsche C., Sahm F., Chavez L., Reuss D.E. (2018). DNA Methylation-based classification of central nervous system tumours. Nature.

[36] Wu Z., Lopes Abath Neto O., Bale T.A., Benhamida J., Mata D., Turakulov R., Abdullaev Z., Marker D., Ketchum C., Chung H.J. (2022). DNA methylation analysis of glioblastomas harboring FGFR3-TACC3 fusions identifies a methylation subclass with better patient survival. Acta Neuropathol..

[37] Wai T., Langer T. (2016). Mitochondrial Dynamics and Metabolic Regulation. Trends Endocrinol. Metab..

[38] Wan Y.Y., Zhang J.F., Yang Z.J., Jiang L.P., Wei Y.F., Lai Q.N., Wang J.B., Xin H.B., Han X.J. (2014). Involvement of Drp1 in hypoxia-induced migration of human glioblastoma U251 cells. Oncol. Rep..

[39] Porper K., Shpatz Y., Plotkin L., Pechthold R.G., Talianski A., Champ C.E., Furman O., Shimoni-Sebag A., Symon Z., Amit U. (2021). A Phase I clinical trial of dose-escalated metabolic therapy combined with concomitant radiation therapy in high-grade glioma. J. Neuro-Oncol..

[40] Yap T.A., Daver N., Mahendra M., Zhang J., Kamiya-Matsuoka C., Meric-Bernstam F., Kantarjian H.M., Ravandi F., Collins M.E., Francesco M.E.D. (2023). Complex I inhibitor of oxidative phosphorylation in advanced solid tumors and acute myeloid leukemia: Phase I trials. Nat. Med..

[41] Shi Y., Lim S.K., Liang Q., Iyer S.V., Wang H.Y., Wang Z., Xie X., Sun D., Chen Y.J., Tabar V. (2019). Gboxin is an oxidative phosphorylation inhibitor that targets glioblastoma. Nature.

[42] Karpel-Massler G., Ishida C.T., Bianchetti E., Shu C., Perez-Lorenzo R., Horst B., Banu M., Roth K.A., Bruce J.N., Canoll P. (2017). Inhibition of Mitochondrial Matrix Chaperones and Antiapoptotic Bcl-2 Family Proteins Empower Antitumor therapeutic Responses. Cancer Res..

[43] Chi A.S., Tarapore R.S., Hall M.D., Shonka N., Gardner S., Umemura Y., Sumrall A., Khatib Z., Mueller S., Kline C. (2019). Pediatric and Adult H3 K27m-Mutant Diffuse Midline Glioma Treated with the Selective DRD2 Antagonist ONC201. J. Neuro-Oncol..

[44] Oltersdorf T., Elmore S.W., Shoemaker A.R., Armstrong R.C., Augeri D.J., Belli B.A., Bruncko M., Deckwerth T.L., Dinges J., Hajduk P.J. (2005). An inhibitor of Bcl-2 family proteins induces regression of solid tumours. Nature.

[45] Philip P.A., Sahai V., Bahary N., Mahipal A., Kasi A., Rocha Lima C.M.S., Alistar A.T., Oberstein P.E., Golan T., Metges J.P. (2024). Devimistat (CPI-613) with Modified Fluorouarcil, Oxaliplatin, Irinotecan, and Leucovorin (Ffx) Versus Ffx for Patients with Metastatic Adenocarcinoma of the Pancreas: The Phase Iii Avenger 500 Study. J. Clin. Oncol..

[46] Pardee T.S., Anderson R.G., Pladna K.M., Isom S., Ghiraldeli L.P., Miller L.D., Chou J.W., Jin G., Zhang W., Ellis L.R. (2018). A Phase I Study of CPI-613 in Combination with High-Dose Cytarabine and Mitoxantrone for Relapsed or Refractory Acute Myeloid Leukemia. Clin. Cancer Res..

[47] Frederick M., Skinner H.D., Kazi S.A., Sikora A.G., Sandulache V.C. (2020). High expression of oxidative phosphorylation genes predicts improved survival in squamous cell carcinomas of the head and neck and lung. Sci. Rep..

[48] Tang L., Wei F., Wu Y., He Y., Shi L., Xiong F., Gong Z., Guo C., Li X., Deng H. (2018). Role of metabolism in cancer cell radioresistance and radiosensitization methods. J. Exp. Clin. Cancer Res..

[49] Valtorta S., Lo Dico A., Raccagni I., Gaglio D., Belloli S., Politi L.S., Martelli C., Diceglie C., Bonanomi M., Ercoli G. (2017). Metformin and temozolomide, a synergic option to overcome resistance in glioblastoma multiforme models. Oncotarget.

[50] Marie S.K., Shinjo S.M. (2011). Metabolism and Brain Cancer. Clinics.

[51] Nayak A.P., Kapur A., Barroilhet L., Patankar M.S. (2018). Oxidative Phosphorylation: A Target for Novel therapeutic Strategies Against Ovarian Cancer. Cancers.

[52] Kim W., Lee S., Seo D., Kim D., Kim K., Kim E., Kang J., Seong K.M., Youn H., Youn B. (2019). Cellular Stress Responses in Radiotherapy. Cells.

[53] Duncan C.G., Killela P.J., Payne C.A., Lampson B., Chen W.C., Liu J., Solomon D., Waldman T., Towers A.J., Gregory S.G. (2010). Integrated genomic analyses identify ERRFI1 and TACC3 as glioblastoma-targeted genes. Oncotarget.

[54] Oudard S., Carpentier A., Banu E., Fauchon F., Celerier D., Poupon M.F., Dutrillaux B., Andrieu J.M., Delattre J.Y. (2003). Phase II study of lonidamine and diazepam in the treatment of recurrent glioblastoma multiforme. J. Neuro-Oncol..

[55] Di Nunno V., Franceschi E., Tosoni A., Gatto L., Lodi R., Bartolini S., Brandes A.A. (2021). Glioblastoma: Emerging Treatments and Novel Trial Designs. Cancers.

[56] Gatto L., Di Nunno V., Franceschi E., Tosoni A., Bartolini S., Brandes A.A. (2022). Pharmacotherapeutic Treatment of Glioblastoma: Where Are We to Date?. Drugs.

[57] Di Nunno V., Franceschi E., Gatto L., Tosoni A., Bartolini S., Brandes A.A. (2023). How to treat histone 3 altered gliomas: Molecular landscape and therapeutic developments. Expert. Rev. Clin. Pharmacol..

[58] Mathupala S.P., Ko Y.H., Pedersen P.L. (2006). Hexokinase Ii: Cancer’s double-edged sword acting as both facilitator and gatekeeper of malignancy when bound to mitochondria. Oncogene.

[59] Milane L., Duan Z., Amiji M. (2011). Development of EGFR-targeted polymer blend nanocarriers for combination paclitaxel/lonidamine delivery to treat multi-drug resistance in human breast and ovarian tumor cells. Mol. Pharm..

[60] Neuzil J., Dong L.F., Rohlena J., Truksa J., Ralph S.J. (2013). Classification of mitocans, anti-cancer drugs acting on mitochondria. Mitochondrion.

[61] Castedo M., Perfettini J.L., Kroemer G. (2002). Mitochondrial apoptosis and the peripheral benzodiazepine receptor: A novel target for viral and pharmacological manipulation. J. Exp. Med..

[62] Homes T.P., Mattner F., Keller P.A., Katsifis A. (2006). Synthesis and In Vitro Binding of N,N-Dialkyl-2-Phenylindol-3-Yl-glyoxylamides for the peripheral benzodiazepine binding sites. Bioorg. Med. Chem..

[63] Solazzo M., Fantappiè O., Lasagna N., Sassoli C., Nosi D., Mazzanti R. (2006). P-Gp Localization in Mitochondria and Its Functional Characterization in Multiple Drug-Resistant Cell Lines. Exp. Cell Res..

[64] Harrington J.S., Ryter S.W., Plataki M., Price D.R., Choi A.M.K. (2023). Mitochondria in health, disease, and ageing. Physiol. Rev..

[65] Heller A., Brockhoff G., Goepferich A. (2012). Targeting drugs to mitochondria. Eur. J. Pharm. Biopharm..

[66] Palmieri F. (2004). The mitochondrial transporter family (Slc25): Physiological and pathological implications. Pflugers Arch..

[67] Szewczyk A., Skalska J., Głab M., Kulawiak B., Malińska D., Koszela-Piotrowska I., Kunz W.S. (2006). Mitochondrial potassium channels: From pharmacology to function. Biochim. Biophys. Acta.

[68] Ishigaki Y., Katagiri H., Yamada T., Ogihara T., Imai J., Uno K., Hasegawa Y., Gao J., Ishihara H., Shimosegawa T. (2005). Dissipating excess energy stored in the liver is a potential treatment strategy for diabetes associated with obesity. Diabetes.

[69] Nübel T., Ricquier D. (2006). Respiration Under Control of Uncoupling Proteins: Clinical Perspective. Horm. Res..

[70] Shang E., Nguyen T.T.T., Westhoff M.A., Karpel-Massler G., Siegelin M.D. (2023). Targeting cellular respiration as a therapeutic strategy in glioblastoma. Oncotarget.

[71] Degli Esposti M. (1998). inhibitors of Nadh-Ubiquinone Reductase: An Overview. Biochim. Biophys. Acta.

[72] Zhao J., Ma X., Gao P., Han X., Zhao P., Xie F., Liu M. (2024). Advancing glioblastoma treatment by targeting metabolism. Neoplasia.

[73] Sesen J., Dahan P., Scotland S.J., Saland E., Dang V.T., Lemarié A., Tyler B.M., Brem H., Toulas C., Cohen-Jonathan Moyal E. (2015). Metformin inhibits growth of human glioblastoma cells and enhances therapeutic response. PLoS ONE.

[74] Ibrahim R.S., Ibrahim S.S., El-Naas A., Koklesová L., Kubatka P., Büsselberg D. (2023). Could metformin and resveratrol support glioblastoma treatment? A mechanistic view at the cellular level. Cancers.

[75] Gammon S.T., Pisaneschi F., Bandi M.L., Smith M.G., Sun Y., Rao Y., Muller F., Wong F., De Groot J., Ackroyd J. (2019). Mechanism-specific pharmacodynamics of a novel Complex-I inhibitor Quantified by imaging reversal of consumptive hypoxia with [(18)F]FAZA PET In Vivo. Cells.

[76] Tsuji A., Akao T., Masuya T., Murai M., Miyoshi H. (2020). Iacs-010759, A Potent inhibitor of glycolysis-deficient hypoxic tumor cells, inhibits mitochondrial respiratory complex I through a unique mechanism. J. Biol. Chem..

[77] Molina J.R., Sun Y., Protopopova M., Gera S., Bandi M., Bristow C., Mcafoos T., Morlacchi P., Ackroyd J., Agip A.A. (2018). An inhibitor of oxidative phosphorylation exploits cancer vulnerability. Nat. Med..

[78] Zhang X., Dang C.V. (2023). Time to hit pause on mitochondria-targeting cancer therapies. Nat. Med..

[79] Zhou Y., Zou J., Zhong X., Xu J., Gou K., Zhou X., Zhou Y., Yang X., Guan X., Zhang Y. (2023). Synthesis and biological evaluation of novel pyrazole amides as potent mitochondrial complex I inhibitors. Eur. J. Med. Chem..

[80] Janku F., Beom S.-H., Moon Y.W., Kim T.W., Shin Y.G., Yim D.-S., Kim G.M., Kim H.S., Kim S.Y., Cheong J.-H. (2022). First-in-human study of IM156, a novel potent biguanide oxidative phosphorylation (OXPHOS) inhibitor, in patients with advanced solid tumors. Investig. New Drugs.

[81] Ralph S.J., Moreno-Sánchez R., Neuzil J., Rodríguez-Enríquez S. (2011). Inhibitors of succinate: Quinone reductase/complex II regulate production of mitochondrial reactive oxygen species and protect normal cells from ischemic damage but induce specific cancer cell death. Pharm. Res..

[82] Kuramoto K., Suzuki S., Sakaki H., Takeda H., Sanomachi T., Seino S., Narita Y., Kayama T., Kitanaka C., Okada M. (2017). Licochalcone a specifically induces cell death in glioma stem cells via mitochondrial dysfunction. FEBS Open Bio.

[83] Kuramoto K., Yamamoto M., Suzuki S., Sanomachi T., Togashi K., Seino S., Kitanaka C., Okada M. (2020). Verteporfin inhibits oxidative phosphorylation and induces cell death specifically in glioma stem cells. FEBS J..

[84] Fiorillo M., Lamb R., Tanowitz H.B., Mutti L., Krstic-Demonacos M., Cappello A.R., Martinez-Outschoorn U.E., Sotgia F., Lisanti M.P. (2016). Repurposing atovaquone: Targeting mitochondrial complex III and oxphos to eradicate cancer stem cells. Oncotarget.

[85] Fang Y., Zhang Z. (2020). Arsenic trioxide as a novel anti-glioma drug: A review. Cell Mol. Biol. Lett..

[86] Chinopoulos C., Seyfried T.N. (2018). Mitochondrial substrate-level phosphorylation as energy source for glioblastoma: Review and hypothesis. ASN Neuro.

[87] Neupane P., Bhuju S., Thapa N., Bhattarai H.K. (2019). ATP synthase: Structure, function and inhibition. Biomol. Concepts.

[88] Stuart S.D., Schauble A., Gupta S., Kennedy A.D., Keppler B.R., Bingham P.M., Zachar Z. (2014). A strategically designed small molecule attacks alpha-ketoglutarate dehydrogenase in tumor cells through a redox process. Cancer Metab..

[89] Nguyen T.T.T., Zhang Y., Shang E., Shu C., Quinzii C.M., Westhoff M.A., Karpel-Massler G., Siegelin M.D. (2020). Inhibition of HDAC1/2 along with TRAP1 causes synthetic lethality in glioblastoma model systems. Cells.

[90] Milane L., Trivedi M., Singh A., Talekar M., Amiji M. (2015). Mitochondrial biology, targets, and drug delivery. J. Control Release.

[91] Nouri K., Feng Y., Schimmer A.D. (2020). Mitochondrial ClpP serine protease-biological function and emerging target for cancer therapy. Cell Death Dis..

[92] Arrillaga-Romany I., Odia Y., Prabhu V.V., Tarapore R.S., Merdinger K., Stogniew M., Oster W., Allen J.E., Mehta M., Batchelor T.T. (2020). Biological activity of weekly ONC201 in adult recurrent glioblastoma patients. Neuro-Oncology.

[93] Cantor E., Wierzbicki K., Tarapore R.S., Ravi K., Thomas C., Cartaxo R., Nand Yadav V., Ravindran R., Bruzek A.K., Wadden J. (2022). Serial H3K27M Cell-free tumor DNA (Cf-tDNA) tracking predicts ONC201 treatment response and progression in diffuse midline glioma. Neuro-Oncology.

[94] Stein M.N., Malhotra J., Tarapore R.S., Malhotra U., Silk A.W., Chan N., Rodriguez L., Aisner J., Aiken R.D., Mayer T. (2019). Safety and enhanced immunostimulatory activity of the DRD2 antagonist ONC201 in advanced solid tumor patients with weekly oral administration. J. Immunother. Cancer.

[95] Guièze R., Liu V.M., Rosebrock D., Jourdain A.A., Hernández-Sánchez M., Martinez Zurita A., Sun J., Ten Hacken E., Baranowski K., Thompson P.A. (2019). Mitochondrial reprogramming underlies resistance to BCL-2 inhibition in lymphoid malignancies. Cancer Cell.

[96] Ramamoorthy M.D., Kumar A., Ayyavu M., Dhiraviam K.N. (2018). Reserpine induces apoptosis and cell cycle arrest in hormone independent prostate cancer cells through mitochondrial membrane potential failure. Anticancer Agents Med. Chem..

[97] Gegg M.E., Cooper J.M., Chau K.Y., Rojo M., Schapira A.H., Taanman J.W. (2010). Mitofusin 1 and Mitofusin 2 are ubiquitinated in A PINK1/Parkin-dependent manner upon induction of mitophagy. Hum. Mol. Genet..

[98] Praefcke G.J., Mcmahon H.T. (2004). The dynamin superfamily: Universal membrane tubulation and fission molecules?. Nat. Rev. Mol. Cell Biol..

[99] Ruiz A., Alberdi E., Matute C. (2018). Mitochondrial division inhibitor 1 (Mdivi-1) Protects neurons against excitotoxicity through the modulation of mitochondrial function and intracellular Ca^2+^ Signaling. Front. Mol. Neurosci..

[100] Sumiyoshi A., Shibata S., Zhelev Z., Miller T., Lazarova D., Aoki I., Obata T., Higashi T., Bakalova R. (2022). Targeting glioblastoma via selective alteration of mitochondrial redox state. Cancers.

[101] Li J., Zhu S., Kozono D., Ng K., Futalan D., Shen Y., Akers J.C., Steed T., Kushwaha D., Schlabach M. (2014). Genome-wide shrna screen revealed integrated mitogenic signaling between dopamine receptor D2 (DRD2) and epidermal growth factor receptor (EGFR) in glioblastoma. Oncotarget.

[102] Przystal J.M., Cianciolo Cosentino C., Yadavilli S., Zhang J., Laternser S., Bonner E.R., Prasad R., Dawood A.A., Lobeto N., Chin Chong W. (2022). Imipridones affect tumor bioenergetics and promote cell lineage differentiation in diffuse midline gliomas. Neuro-Oncology.

[103] Venneti S., Kawakibi A.R., Ji S., Waszak S.M., Sweha S.R., Mota M., Pun M., Deogharkar A., Chung C., Tarapore R.S. (2023). Clinical efficacy of ONC201 in H3K27M-mutant diffuse midline gliomas is driven by disruption of integrated metabolic and epigenetic pathways. Cancer Discov..

[104] Prabhu V.V., Morrow S., Rahman Kawakibi A., Zhou L., Ralff M., Ray J., Jhaveri A., Ferrarini I., Lee Y., Parker C. (2020). ONC201 and Imipridones: Anti-cancer compounds with clinical efficacy. Neoplasia.

[105] Wierzbicki K., Ravi K., Franson A., Bruzek A., Cantor E., Harris M., Homan M.J., Marini B.L., Kawakibi A.R., Ravindran R. (2020). Targeting and therapeutic monitoring of H3k27m-mutant glioma. Curr. Oncol. Rep..

[106] Allen J.E., Krigsfeld G., Mayes P.A., Patel L., Dicker D.T., Patel A.S., Dolloff N.G., Messaris E., Scata K.A., Wang W. (2013). Dual inactivation of Akt and ERK by TIC10 Signals Foxo3a nuclear translocation, trail gene induction, and potent antitumor effects. Sci. Transl. Med..

[107] Kline C.L., Van Den Heuvel A.P., Allen J.E., Prabhu V.V., Dicker D.T., El-Deiry W.S. (2016). ONC201 kills solid tumor cells by triggering an integrated stress response dependent on ATF4 Activation by Specific eIF2α kinases. Sci. Signal..

[108] Bonner E.R., Waszak S.M., Grotzer M.A., Mueller S., Nazarian J. (2021). Mechanisms of imipridones in targeting mitochondrial metabolism in cancer cells. Neuro-Oncology.

[109] Stein M.N., Bertino J.R., Kaufman H.L., Mayer T., Moss R., Silk A., Chan N., Malhotra J., Rodriguez L., Aisner J. (2017). First-in-human clinical trial of oral ONC201 in patients with refractory solid tumors. Clin. Cancer Res..

[110] Arrillaga-Romany I., Chi A.S., Allen J.E., Oster W., Wen P.Y., Batchelor T.T. (2017). A Phase 2 Study of the First Imipridone ONC201, A selective DRD2 antagonist for oncology, administered every three weeks in recurrent glioblastoma. Oncotarget.

[111] Wagner J., Kline C.L.B., Baumeister M., El-Deiry W.S. (2016). Abstract 3000: Intra-tumoral accumulation of NK1.1/CD3+ cells and anti-metastasis effects of dose-intensified ONC201 in tumor-bearing mice. Cancer Res..

[112] Hall M.D., Odia Y., Allen J.E., Tarapore R., Khatib Z., Niazi T.N., Daghistani D., Schalop L., Chi A.S., Oster W. (2019). First clinical experience with drd2/3 antagonist ONC201 in H3 K27M-mutant pediatric diffuse intrinsic pontine glioma: A case report. J. Neurosurg. Pediatr..

[113] Tanrıkulu B., Yaşar A.H., Canpolat C., Çorapçıoğlu F., Tezcanli E., Abacioglu U., Danyeli A.E., Özek M.M. (2023). Preliminary findings of german-sourced ONC201 treatment in H3K27 altered pediatric pontine diffuse midline gliomas. J. Neuro-Oncol..

[114] Arrillaga-Romany I., Gardner S.L., Odia Y., Aguilera D., Allen J.E., Batchelor T., Butowski N., Chen C., Cloughesy T., Cluster A. (2024). ONC201 (Dordaviprone) in recurrent H3 K27M-mutant diffuse midline glioma. J. Clin. Oncol..

[115] Carter J.L., Hege K., Kalpage H.A., Edwards H., Hüttemann M., Taub J.W., Ge Y. (2020). Targeting mitochondrial respiration for the treatment of acute myeloid leukemia. Biochem. Pharmacol..

[116] Jackson E.R., Duchatel R.J., Staudt D.E., Persson M.L., Mannan A., Yadavilli S., Parackal S., Game S., Chong W.C., Jayasekara W.S.N. (2023). ONC201 in combination with paxalisib for the treatment of H3K27-altered diffuse midline glioma. Cancer Res..

[117] Nguyen T.T.T., Zhang Y., Shang E., Shu C., Torrini C., Zhao J., Bianchetti E., Mela A., Humala N., Mahajan A. (2020). Hdac inhibitors elicit metabolic reprogramming by targeting super-enhancers in glioblastoma models. J. Clin. Investig..

[118] Nguyen T.T.T., Shang E., Schiffgens S., Torrini C., Shu C., Akman H.O., Prabhu V.V., Allen J.E., Westhoff M.A., Karpel-Massler G. (2022). Induction of synthetic lethality by activation of mitochondrial ClpP and inhibition of HDAC1/2 in glioblastoma. Clin. Cancer Res..

[119] Guo J., Xue Q., Liu K., Ge W., Liu W., Wang J., Zhang M., Li Q.Y., Cai D., Shan C. (2019). Dimethylaminomicheliolide (DMAMCL) suppresses the proliferation of glioblastoma cells via targeting Pyruvate Kinase 2 (PKM2) and rewiring aerobic glycolysis. Front. Oncol..

